# A primer on systematic reviews in toxicology

**DOI:** 10.1007/s00204-017-1980-3

**Published:** 2017-05-13

**Authors:** Sebastian Hoffmann, Rob B. M. de Vries, Martin L. Stephens, Nancy B. Beck, Hubert A. A. M. Dirven, John R. Fowle, Julie E. Goodman, Thomas Hartung, Ian Kimber, Manoj M. Lalu, Kristina Thayer, Paul Whaley, Daniele Wikoff, Katya Tsaioun

**Affiliations:** 1 Evidence-Based Toxicology Collaboration at Johns Hopkins Bloomberg School of Public Health, Baltimore, MD USA; 2 SYRCLE (SYstematic Review Centre for Laboratory Animal Experimentation), Department for Health Evidence (section HTA), Radboud Institute for Health Sciences, Radboud University Medical Center, Nijmegen, The Netherlands; 3 American Chemistry Council, Washington, DC USA; 4 Norwegian Institute of Public Health, Oslo, Norway; 5 Science to Inform, LLC, Pittsboro, NC USA; 6 Gradient, Cambridge, MA USA; 7 Center for Alternatives to Animal Testing at Johns Hopkins Bloomberg School of Public Health, Baltimore, MD USA; 8 University of Manchester, Manchester, UK; 9 Clinical Epidemiology Program, Regenerative Medicine Program, Department of Anesthesiology and Pain Medicine, The Ottawa Hospital Research Institute, University of Ottawa, Ottawa, Canada; 10 US Environmental Protection Agency, Washington, DC USA; 11 Lancaster Environment Centre, Lancaster University, Lancaster, UK; 12 ToxStrategies, Asheville, NC USA; 13 seh consulting + services, Paderborn, Germany

**Keywords:** Systematic review, Evidence-based toxicology, Narrative review, Evidence synthesis, Review steps

## Abstract

Systematic reviews, pioneered in the clinical field, provide a transparent, methodologically rigorous and reproducible means of summarizing the available evidence on a precisely framed research question. Having matured to a well-established approach in many research fields, systematic reviews are receiving increasing attention as a potential tool for answering toxicological questions. In the larger framework of evidence-based toxicology, the advantages and obstacles of, as well as the approaches for, adapting and adopting systematic reviews to toxicology are still being explored. To provide the toxicology community with a starting point for conducting or understanding systematic reviews, we herein summarized available guidance documents from various fields of application. We have elaborated on the systematic review process by breaking it down into ten steps, starting with planning the project, framing the question, and writing and publishing the protocol, and concluding with interpretation and reporting. In addition, we have identified the specific methodological challenges of toxicological questions and have summarized how these can be addressed. Ultimately, this primer is intended to stimulate scientific discussions of the identified issues to fuel the development of toxicology-specific methodology and to encourage the application of systematic review methodology to toxicological issues.

## Preamble

Evidence-based approaches are received growing attention in toxicology due to their potential to improve the field’s transparency, objectivity, consistency and reproducibility, and to inform regulatory decisions and policy more effectively (Guzelian et al. 2005; Hoffmann and Hartung 2006; Schreider et al. 2010; Woodruff and Sutton 2014; Thayer et al. 2014; National Toxicology Program 2015; Stephens et al. 2016). By analogy to evidence-based medicine (EBM), the umbrella term evidence-based toxicology (EBT) has been coined to group all approaches intended to implement more effectively evidence-based principles in toxicology in general, and in toxicological decision making in particular. Such approaches include inter alia the establishment and universal use of a common ontology, justified design and rigorous conduct of studies, consistently structured and detailed reporting of experimental evidence, structured frameworks for evidence synthesis that characterize confidence in the evidence, probabilistic uncertainty and risk assessment, and the development of synthesis methodology to integrate evidence from diverse streams, e.g., from human observational studies, animal studies, in vitro studies and in silico/mathematical modeling.

The core evidence-based tool is the systematic review. Much attention has been focused on the application of systematic review methodology to toxicological questions in line with the efforts of government institutions from both sides of the Atlantic, such as the European Food Safety Authority (EFSA 2010) and the US National Toxicology Program’s (NTP) (Birnbaum et al. 2013). The work of these organizations has triggered the adoption and adaptation of systematic review approaches as a tool for conducting evidence-based assessments (EFSA 2010; Rooney et al. 2014; National Research Council 2014). As these initiatives are focused on the requirements and mandates of the respective institutions, the Evidence-Based Toxicology Collaboration (EBTC) and its stakeholders have identified the need to build on these recent developments and to provide a general introduction to systematic reviews for the broader toxicology community.

**Fig. 1:**
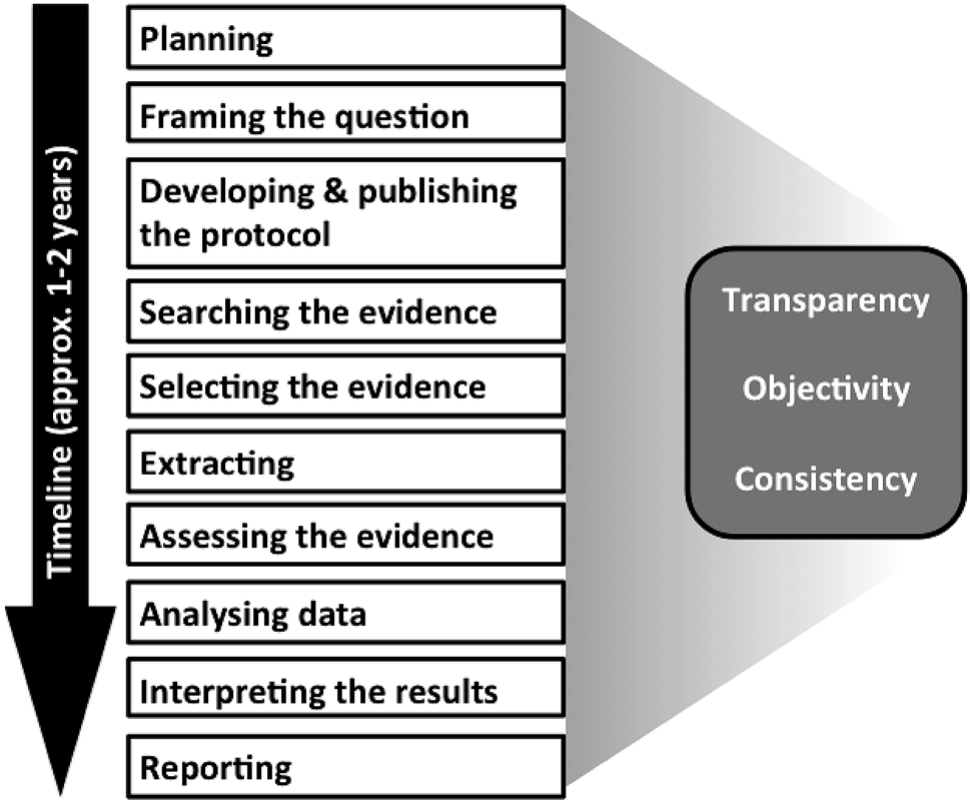
Steps of a systematic review

Building on calls for systematic reviews, e.g., by Stephens et al. (2013), Silbergeld and Scherer (2013) and Whaley et al. (2015), this primer is intended to serve as a starting point for toxicologists interested in understanding or conducting systematic reviews. While not a manual or handbook, sufficient detail is provided to allow basic understanding of the principles, process, and resources required to conduct a systematic review. The review process has been broken down into ten sequential steps identified by Stephens et al. (2016), which were used to structure this guidance (Fig. 1). The application of systematic reviews in toxicology is still in its early days and will continue to evolve. In particular, many methodological aspects are being discussed and consensus still needs to be reached. Consequently, this primer attempts to summarize existing proposals by identifying commonalities to introduce a common terminology (see Glossary), and to highlight the challenges ahead.

Historically, reviews in toxicology have been predominantly narrative in approach, whereby an expert uses literature to summarize a particular field, or attempts to address a specific research question, for example, regarding the potential toxicity of a chemical or drug for humans. A narrative review typically uses an implicit process to compile evidence to support the statements being made in the review. The reader often cannot tell how the available literature was identified, selected and compiled, why some studies were given more weight than others, and how the evidence was summarized to arrive at conclusions. It is often uncertain whether the author of a narrative review selectively cited reports that reinforced his or her preconceived ideas, or promoted specific views of a topic. Also, a quantitative summary of the literature is often absent in a narrative review.

Overall, these issues increase the risk that a review will produce misleading results through selective use and/or interpretation of the available evidence, and transmission of bias and error in the reviewed evidence to the final summary results. Lack of transparency in reporting of review methods can make it very difficult for the reader to detect such shortcomings. Given the numerous sources of potential bias, and the lack of transparency and methodological rigor, traditional “narrative” toxicological reviews are at an increased risk of being biased and often cannot be independently reproduced. This makes it difficult to confirm a review’s conclusions and runs the risk of misdirecting future research. In worst cases, risk management decisions based ostensibly on the same evidence base may differ significantly, as summarized by Whaley et al. (2015) for Bisphenol A or by Rudén (2001) for trichloroethylene, leading to a variety of issues, including uncertainty for all stakeholders. This undermines trust in decision makers’ and impedes consumers’ decision making, potentially jeopardizing public health. It should be noted, however, that notwithstanding their shortcomings for purposes such as summarizing toxicological knowledge or informing decision making, narrative reviews have their place in toxicology, e.g., when an expert view on a topic is needed or when time to make a decision is limited, as long as the nature of the review is made explicit. Table 1, adapted from de Vries et al. (2014), summarizes how various features differ between narrative and systematic reviews. While this summary provides a general overview and is in most features a relative comparison of the review types, it demonstrates that the rigor of systematic reviews often requires increased time and resources.

**Table 1:** Some differences between systematic and narrative reviews

Feature	Narrative review	Systematic review
Research question	Broad and informal (often not explicitly specified)	Specified and specific
Literature sources and search	Usually not specified	Comprehensive sources (more than one database) and explicit search strategy
Study selection	Usually not specified	Explicit selection criteria
Quality assessment of included studies	Usually not present or informal (not explicitly specified)	Critical appraisal on the basis of explicit criteria
Synthesis	Often a qualitative summary	Qualitative and sometimes also a quantitative summary (meta-analysis)
Time	Months^a^	>1 year (usually)
Required expertise	Science	Science, systematic review, literature searches, data analysis (including meta-analysis)
Costs	Low^a^	Moderate to high

Note that these are just generalized estimates and true costs are likely to be variable for both narrative and systematic reviews

^a^Narrative reviews of authorities may take years, which are associated with high costs

Historically, clinical research reviews were expert-written narrative reviews as well, before the advent of the evidence-based medicine/healthcare (EBM/EBHC) movement. This movement established systematic review methodology as the best practice for summarizing all available evidence bearing on a research question. The need for reproducible, transparent, and comprehensive syntheses of the ever-growing volume of medical evidence triggered the development of increasingly rigorous approaches to review question formulation, literature search, evidence selection, and evidence integration. The field of clinical systematic reviews has grown into a large discipline with offshoot products, such as PRISMA (Preferred Reporting Items for Systematic Reviews and Meta-Analyses; www.prisma-statement.org), which is a guideline for reporting systematic reviews. In addition, working groups such as GRADE (Grading of Recommendations Assessment, Development and Evaluation) have developed to better understand and interpret the results of systematic reviews. The Cochrane group (http://www.cochrane.org), previously known as the Cochrane Collaboration, has played an instrumental role in fostering the continued development of systematic review methodologies since 1993. The *Cochrane Handbook for Systematic Reviews of Interventions* provides detailed guidance and instructions for conducting systematic reviews in a medical context (Higgins and Green 2011). Cochrane is also drafting a handbook for systematic reviews of diagnostic test accuracy (DTA) that adapts the evidence-based approaches to the challenges associated with evaluating diagnostic testing. Many of the methods in both handbooks are widely applicable, so they can be directly adopted for conducting systematic reviews in non-clinical areas, such as toxicology, while other methods need to be adapted for the toxicology context.

However, it is important to mention the specific differences between toxicology and clinical research, and the unique challenges associated with the application of this framework to toxicological questions (Wikoff and Britt 2016). These include multiple evidence streams and the challenges of their integration, multiple animal species (and strains), multiple outcomes and endpoints that characterize hazards, exposures to complex mixtures and the frequent lack of human data engendering the need to extrapolate from other species to human outcomes. Further, the objectives in a toxicological review often involve the evaluation of adversities as compared to clinical interventions. These complexities make it clear that the process developed for systematically reviewing randomized clinical trials for medical interventions, while serving as a foundational framework, will have to be substantially adapted to be applicable and useful in toxicology.

This primer relies heavily on existing authoritative documents, such as guidance and handbooks. We considered documents that were known to us, identified by a (non-systematic) internet search or included in the references of already identified guidance documents. This process yielded eight guidance documents (Table 2). We distilled the information from them by extracting the relevant details for each of the ten systematic review steps in our framework, focusing primarily on the Cochrane Handbook (Higgins and Green 2011), the EFSA guidance (EFSA 2010), the OHAT guidance (National Toxicology Program 2015) and the Collaboration for Environmental Evidence guidance (CEE 2013). The guidance provided by the Institute of Medicine (IOM 2011), Agency for Healthcare Research and Quality (AHRQ 2014) and Centre for Reviews and Dissemination (CRD 2009) is largely similar to the Cochrane Handbook, but with special focus on the needs and requirements of the individual organizations. The National Research Council’s (NRC’s) analysis of the US Environmental Protection Agency’s (EPA) Integrated Risk Information System (IRIS) process reviewed inter alia current methods for evidence-based reviews. Other guidances, such as the systematic review methodology of the Navigation Guide (Woodruff and Sutton 2014) and the recommendations for systematic review and evidence integration by the Texas Commission on Environmental Quality (TCEQ 2014), are generally in agreement with the guidances considered here. In many cases, the approaches put forward in the individual guidance documents were similar to each other and consequently to that presented here. However, we also found some specific issues, on which not all guidance documents were aligned. In these cases, we either choose to present the approach proposed by the majority or describe the divergent views.

**Table 2:** The guidance documents that were considered

Title	Version/publication date	Authors/editors	Link
Application of systematic review methodology to food and feed safety assessments to support decision making	2010	European food safety agency	http://www.efsa.europa.eu/de/efsajournal/doc/1637.pdf
Cochrane handbook for systematic reviews of interventions	5.1.0 (2011)	Higgins JPT, Green S	http://www.cochrane.org/handbook
Finding what works in healthcare: standards for systematic reviews	2011	Institute of medicine (IOM): Committee on standards for systematic reviews of comparative effectiveness research	http://www.nap.edu/catalog/13059/finding-what-works-in-health-care-standards-for-systematic-reviews
Guidelines for systematic reviews in environmental management	4.2 (March 2013)	Collaboration for environmental evidence	http://environmentalevidence.org/wp-content/uploads/2014/06/Review-guidelines-version-4.2-final.pdf
Handbook for conducting a literature-based health assessment using OHAT approach for systematic review and evidence integration	2015	National toxicology program OHAT	https://ntp.niehs.nih.gov/ntp/ohat/pubs/handbookjan2015_508.pdf
Methods guide for effectiveness and comparative effectiveness reviews	2014	Agency for healthcare research and quality	http://effectivehealthcare.ahrq.gov/ehc/products/60/318/CER-Methods-Guide-140109.pdf
Review of EPA’s integrated risk information system (IRIS) process	2014	National research council (NRC)	http://www.nap.edu/catalog/18764/review-of-epas-integrated-risk-information-system-iris-process
Systematic reviews: CRD’s guidance for undertaking systematic reviews in healthcare	2009	Centre for reviews and dissemination (CRD), University of York, UK)	https://www.york.ac.uk/media/crd/Systematic_Reviews.pdf

Reliance on these guidance documents should not be interpreted as an endorsement of any particular approach at this point in time as the field and methodology are continuing to develop. In addition, it should be noted that we deliberately did not conduct a systematic review on this topic. As the purpose was rather to provide a survey of available guidance and characterize the key components in the conduct of a systematic review and in doing so, highlight some of the challenges in applying existing frameworks to toxicological questions, we considered a narrative approach to be more suitable.

To date, the application of systematic review methodology to toxicological issues has focused primarily on questions regarding the impact of chemicals on human health, e.g., for perfluorooctanoic acid (PFOA) (Johnson et al. 2014; Koustas et al. 2014) or metals (Navas-Acien et al. 2007; Meyer-Baron et al. 2009). However, many other toxicological questions are suitable for systematic review. Examples are, the risk associated with a specific exposure (Tsuji et al. 2014), the (eco-)toxicity of mixtures of substances (Cedergreen 2014), the relevance of a toxicity biomarker (Dello et al. 2013), the assessment of new toxicological test methods, the determination of toxicological mechanism, e.g., in the frame of the Adverse Outcome Pathways (AOP) approach, the status of technology development relevant for toxicological questions (de Vries et al. 2013), and the evaluation of risks to human health posed by a chemical under specific regulations, such as the Regulation, Evaluation and Authorisation of Chemicals (REACH) in Europe (Whaley et al. 2015). Note that the examples given here claimed to be systematic reviews. However, it is important to note that our citing of those examples here does not imply that these publications met the systematic review criteria in all cases. Indeed, an increasing number of publications claim to be systematic reviews, but upon a closer examination, fail to meet basic criteria such as the production of a review protocol, the documentation of the literature search, or the appraisal of studies (Haddaway et al. 2017).

The application of many of the individual framework steps in a systematic review, e.g., the approach and reporting for evidence search, will be very similar for any review question. However, as outlined above, toxicological reviews have specific challenges that call for adaptation of the established systematic review methodology, especially the potential diversity of evidence. This primer summarizes the systematic review process and methods in a way intended to be primarily useful for assessing the toxicity of chemical substances. However, it will also be useful when considering other types of review questions, such as the assessment of test methods for hazard identification, characterization and the elucidation of a toxicological mechanism as well as the establishment of health-based toxicity values. In addition, it identifies some important methodological and structural challenges we are currently facing. It is anticipated that this primer will serve as a helpful introduction for those unfamiliar with the tool, as well contribute to a common acceptance of the rigor involved with the conduct of systematic reviews in support of toxicological assessments.

1. Planning
  Motivation
  Scoping
  Review team
  Advisory group
  Sponsors

Because minimizing bias is a guiding principle of systematic reviews, even the initial planning should be conducted as rigorously, objectively, and transparently as possible. This step may involve iterative consideration of sponsor and stakeholder needs, scoping of the topic—including considerations of feasibility, and input and participation from a multidisciplinary team sharing a variety of roles.

The *motivation* to conduct a systematic review should be documented to provide a summary of what is known on a given topic, e.g., to summarize a large amount of evidence, to explore reasons for inconsistency in the results of studies, resolve controversies or uncertainty about what the existing evidence is demonstrating, or to identify data gaps. Once the plan to conduct a review assumes shape, it has to be decided which type of review to perform. In some cases, a narrative approach may be chosen for any of a variety of reasons, such as limited available time or resources, limited data or to express an expert opinion. However, if the goal is to provide an objective and comprehensive summary of the evidence on a certain topic, a systematic review approach should be conducted. Table 1 (see “Preamble”) suggests that it is conceivable to adopt a mixed review approach that addresses some features in the manner of a narrative review and others in the manner of a systematic review. It is emphasized, however, that only a review conducted systematically in all steps is a systematic review. To avoid improper use of terminology, a mixed review approach should not be called a systematic review.

Various motivations exist to conduct a systematic review in toxicology. It is conceivable that the motivated party is researchers, who conduct systematic reviews to answer questions in their specific field of interest, or governmental, nonprofit or commercial organizations, which may conduct systematic reviews themselves or sponsor them. In the frameworks that have been created by agencies, including the NTP and EFSA, the motivation is driven by the respective public health mandates and needs of the conducting entity. Whether conducted by an agency or not, a systematic review may seek to clarify the health effects of an evidence-rich chemical. In other cases, a systematic review may be undertaken when evidence is scarce to identify data gaps or to assess the accuracy of a toxicological test method. Given that the systematic review framework is still an emerging practice in toxicology, it is also possible in these early days that a systematic review may be conducted, in part, to explore the proper translation of this methodology to the toxicological arena.

The questions addressed by systematic reviews should be meaningful to relevant stakeholders. Once the question and objective(s) for a systematic review have been at least roughly formulated, an effort should be made to make certain that no systematic review of sufficient quality and timeliness already exists. If a systematic review addressing a similar question is currently in progress, the results should be awaited prior to considering the undertaking of another one. In this regard, a registry of ongoing or completed systematic reviews in toxicology would be helpful. The PROSPERO database (http://www.crd.york.ac.uk/PROSPERO/), may serve as such a registry as the inclusion criteria have been expanded to systematic reviews with a health-related outcome. Other options are available, such as the publication of protocols for systematic reviews of laboratory animal studies offered by SYRCLE (SYstematic Review Centre for Laboratory animal Experimentation).

While not necessarily required, *scoping* the literature on the topic could be helpful in assessing the need for a systematic review. This approach is particularly useful in fields where little is known regarding the current state of the literature and previous systematic reviews have not been performed. Scoping may range from a simple non-systematic search in one or two databases to a more formalized, resource intensive scoping review (described in Levac et al. 2010 and in Peters et al. 2015). If a more formalized approach is adopted, it is recommended to consult with or involve a trained information specialist in conducting comprehensive literature searches for systematic reviews (McGowan and Sampson 2005). The findings of a scoping exercise may reveal that the question has already been adequately addressed or may confirm that better understanding of the evidence could provide clarity. A scoping search can inform the planning process by revealing important details such as the expertise required, the stakeholders that have interest in the topic, and the resources needed. Scoping may be conducted before or after a review team is formed, but the approach used should be transparent and objective.

A *review team* should be created and the roles and responsibilities of the team members should be defined during the planning phase. This team should be multidisciplinary and combine appropriate expertise and experience to conduct the systematic review. It should include expertise on (1) the topic, (2) systematic review methodology, (3) literature search and retrieval, and if required, (4) quantitative methods and statistics. Two or more members of the team should collaborate to allow cross-checking of essential systematic review steps, many of which require parallel work independently conducted. The team should establish a leader who understands the task in detail and is skilled in facilitating multidisciplinary projects. Among the first tasks of the review team should be detailed planning of required resources, distribution of tasks and planning of the time frame. Furthermore, the team should engage with expected users of the review’s results and diverse stakeholders to collect their input. Bias in the review team should be minimized and disclosed. Members should be independent of parties with potential conflicts of interest. All members should complete a formal conflict of interest statement, e.g., using the International Committee of Medical Journal Editors (ICMJE) Conflict of Interest form (COI). (available at http://www.icmje.org), which may need to be re-visited throughout the review, e.g., using a COI management plan.

An *advisory group* that includes representation of relevant stakeholders, especially potential users of the systematic review outcome with appropriate interests, skills and commitment, should be considered. The availability of an advisory group may prove especially valuable in supporting the review team by informing key decisions, particularly those that arise from the need to adapt general systematic review methodology to the needs of toxicology. In addition, the advisory group can help interpret and disseminate results, and collect stakeholder and user input.

If not yet available, potential *sponsors* that provide financial resources may be approached. Sponsors of systematic reviews can be governmental, nonprofit or commercial, noting, however, that in the clinical field, some (e.g., Cochrane) prohibit commercial sponsors. They should not interfere with the independence of the review team. While allowed to give direction in the very early stages of a systematic review, sponsors should not exert any influence once the (broad) review question is defined. However, input on the scope as well as oversight of the review to ensure progress and timeliness should be allowed. All sponsorships should be acknowledged. It is advisable that the motivation of the sponsor to support a specific systematic review should be made known to all parties involved. Best practices regarding sponsorship are likely to evolve with the acceptance and application of systematic reviews in toxicology.

By the end of the planning stage, the decision to conduct the review will have been confirmed (or not). The resources and the timeframe will have been established, and the review team and advisory group will be in place.

For toxicological systematic reviews, the major challenge in the planning phase will be to compose a skilled review team. In particular, systematic review experience among toxicologists is scarce. Until sufficient systematic review capacity is built in toxicology, clinical or pre-clinical systematic review experts may need to be engaged. Also, the issue of sponsorship is delicate. While such sponsorship may be essential to progress in this field, this needs to be weighted against the potential bias introduced. Clear boundaries for sponsor interaction need to be established very early in the review process and detailed documentation of the sponsor’s role may present a solution.

2. Framing the question
  Review question(s)
  Components (PICO/PECO)
  Modifications

Once the need for a systematic review has been established, that need should be translated into the *review question(s)* for conducting the review. Framing the review question(s) is a crucial step in a systematic review.

When reviewing clinical questions/interventions, the process of formulating a review question follows a structured framework that consists of a few essential *components*. One of the approaches for formulating systematic review questions used in medicine is captured in the *PICO* framework. This framework calls for the systematic review question to address the Population/participants, the Intervention, the Comparison or Control, and, if considered relevant, the Outcome. In addition, the review question may also specify the types of studies to be considered, e.g., randomized clinical trials, and may include the (clinical) setting, which is sometimes reflected by amending the framework to PICOTS.

In general, this framework should also be applicable to the toxicological context; it has been adapted to exposure-related review questions as *PECO*, replacing the Intervention component with Exposure, defined by exposure conditions, e.g., a substance or radiation, the route (e.g., oral, dermal, intravenous), the duration (ranging from once (acute) to daily for a lifetime (chronic)) and the relevant exposure range. Appropriate definition of the exposure component is essential for the relevance of the review results for public health protection. An example of a PECO for a chemical health effect is to investigate if chronic oral exposure to chemical X (exposure) induces health effect Y (outcome) in adult rats (population) as compared to not exposed adult rats (control). The population should clearly define the evidence stream(s) and the subjects considered (e.g., adult, juvenile/children, pregnant, healthy, diseased, etc.).

For systematic reviews assessing the accuracy of a test method compared with another, one would need to specify an index test and a comparator test. Note that not all components are relevant to every systematic review and that specific questions may require different components. Regardless of specifics, framework components are a general requirement and are specified in the systematic review protocol. The review team should plan sufficient time for framing the review question, including the generation of the associated rationale and context, and possibly for iterative modifications, as decisions made during problem formulation have significant impact on the scope and form of the systematic review.

Examples of toxicological review questions addressed in recent systematic reviews or intended for systematic reviews include:

- What is the effect of exposure to fluoride used as additive for water fluoridation, compared to vehicle-only treatment, on neurobehavioral outcomes in whole non-human mammalian animals? (National Toxicology Program 2016).
- What is the epidemiological evidence for an association of low-level arsenic exposure in drinking water with cardiovascular disease? (Tsuji et al. 2014).
- What is the animal and human evidence for the usefulness of ophthalmate as a biomarker for oxidative stress and hepatic glutathione homeostasis? (Dello et al. 2013).
- What is the association between intake of isoflavones from food supplements and adverse effects on the three target organs (mammary gland, uterus and thyroid) in peri- and post-menopausal women? (EFSA Panel ANS 2015).
- Is developmental exposure to air pollution associated with autism spectrum disorder? (Lam et al. 2016).
- For healthy adults, is caffeine intake above 400 mg/day, compared to intakes of 400 mg/day or less, associated with adverse effects on cardiovascular outcomes? (PROSPERO 2015: CRD42015026673; available from http://www.crd.york.ac.uk/PROSPERO/display_record.asp?ID=CRD42015026673).

While secondary review question(s) may be addressed, a clear single primary review question should drive the formulation of the review. Because this question will be the systematic review’s guiding element and principal goal, defining it precisely and appropriately is of crucial importance; the entire review team should be involved in the process. A properly framed review question will facilitate all the review’s subsequent steps, including the definition of the eligibility criteria and the literature search, how the evidence/data will be collected, and how the results will be presented and integrated. In particular, the question should help define the criteria for the inclusion and exclusion of research studies in a way that ensures that all relevant evidence is included to answer a particular question. For example, the review question could focus on a specific study type, such as chronic toxicity studies in animals, and would exclude any other study type, such as acute or sub-acute toxicity studies. An example of a question about a specific hazard of a substance would be ‘What is the current evidence from animal studies that substance X compared to substance Y (e.g., vehicle or no treatment) can induce effect Y?’

Once the review question has been formulated, it can be modified if more detailed insight into the topic demands it. However, all *modifications* should be well documented and justified, agreed to by the entire review team, eventually approved by the advisory group and reported, preferably, both in a protocol and in the final publication. Any introduction of bias, such as modifying the review question after data extraction should be strictly avoided. Modifications to the question during protocol development may be indicated, for example, when the scope of the review question proves to be too narrow or wide, the study type(s) to be considered need to be restricted or extended, or the outcome of interest is too specific or nonspecific (e.g., developmental effects vs malformations vs delayed ossification).

A major challenge will be to frame questions in a way that they are amenable to systematic reviews. The initiating problem might be too broad for a single systematic review, e.g., to perform a human health risk assessment for chemical X. In this case, the broad question needs to be distilled into various smaller PECO questions allowing for a systematic review, for example, focusing on specific risk, a single human health endpoints and/or a limited number of outcomes/endpoints.

3. Developing and publishing the protocol
  Protocol development
  Essential steps
  Protocol publication and registration

Once the review question has been defined, the protocol needs to be developed. *Protocol development* is often an iterative process. To minimize bias, the protocol should specify the methods to conduct the systematic review in such detail that the review could be independently reproduced. This reduces the potential for introducing bias, because the process is defined without detailed knowledge of the evidence. As the review team’s understanding of the topic evolves, issues may arise that make the need for adjustments to the protocol apparent. To ensure the transparency of any protocol modifications, the review team needs to document and justify them. Developing the protocol may require regular communication within the review team and with the advisory group. Standardized protocol formats are available for clinical systematic reviews (e.g., in Higgins JPT and Green S (2011)) and for animal intervention studies (e.g., by de Vries et al. 2015), but not for systematic reviews of toxicological issues.

The protocol should address how the systematic review’s *essential steps* will be carried out to maximize transparency and consistency and to reduce biases. Special care must be taken to avoid the possibility of having biases introduced by the review team, e.g., simply by being aware of this potential bias or by requesting the support of the advisory group. At least some members of the team are by definition knowledgeable about the review topic and may therefore have predetermined opinions and expectations. In addition to presenting the review question to be addressed in detail, including background information and any secondary questions, and administrative information, the protocol should specify (see e.g., PRISMA-P (Moher et al. 2015):

- The literature search strategy, including the databases and other sources to be searched, the languages to be considered, the publication period to be covered as well as database-specific search syntax.
- The inclusion/exclusion criteria detailing how the studies/hits identified during the search, which can be in the thousands, will be screened for relevance, and a description of the process how these will be applied. Usually, at least two reviewers screen the studies independently in duplicate, initially based on title and abstracts, and later on full texts.
- The data to be extracted and the process of data extraction, including an explanation of how it will be retrieved from disparate data sources. This should involve at least two trained extractors that abstract data independently in duplicate using standardized forms/templates.
- The criteria that will be used to assess each evaluated study’s quality, including internal validity/risk of bias, i.e., the degree to which a result of a study is likely to be true and free of bias, such as selection bias, performance bias or detection bias, but also other quality aspects, e.g., related to exposure, and a description of the process how these will be applied. Usually, at least two reviewers screen the studies independently in duplicate. Instructions for assessing the entire body of evidence’s risk of bias should also be included.
- How the data will be summarized and synthesized relative to develop conclusions, which may or may not include a quantitative analysis, e.g., a meta-analysis. Here, it is helpful to anticipate the likely data types to be encountered, e.g., ordinal or dichotomous, one- or multi-dimensional, and to determine how the (summary) data will be represented when the data are synthesized.
- The process of determining confidence in the final result distilled out of the included studies considering aspects such as precision, consistency, directness, magnitude, dose–response relationship, publication bias and aspects of quality, including external validity, and internal validity/risk of bias).

Once the review team considers the protocol as complete, it should be made publicly available. This allows interested parties that were not involved or consulted in the preparation of the systematic review, such as additional experts or stakeholders, to provide constructive input at this stage that still allows the team to accommodate suggestions. The review team should be, within limits, responsive to the comments received. Additionally, *protocol publication* or registration serves as documentation of a priori decisions—a critical component of systematic reviews. For example, it safeguards against introduction of bias via changing methods part-way through the review process, as it requires justifying the points in which the actual report of results deviates from the protocol. Protocols can be submitted at various stages, ranging from the very initial stages of problem formulation, but prior to the completion of data extraction. Several venues for protocol publication or registration are available. Aside from PROSPERO, HAWC (Health Assessment Workspace Collaborative), which is designed to facilitate development of human health assessments of chemicals, provides an opportunity for authors to document and make protocols publicly available. Other sources to publish protocols include: Open Science Framework (www.osf.io), CAMARADES for animal systematic reviews (http://www.dcn.ed.ac.uk/camarades/), or any electronic repository that is publicly available and searchable (e.g., UOttawa repository—https://www.ruor.uottawa.ca/).

While a central site for registration of toxicological systematic review protocols is a long-term goal, it will be a challenge in these early days to publish protocols that are highly visible to stakeholders and interested parties, unless they are conducted by governmental agencies with established dissemination channels. In addition, ways to ensure timely communication of protocol publication to the intended audiences need to be established, e.g., through presentation at relevant conferences.

4. Searching the evidence
  Design of a search strategy
  Implementation of the search
  Biases
  Sources to search
  Documentation

The literature search is at the heart of systematic review. It needs to be sensitive enough that it does not inadvertently exclude evidence, which is relevant to the review question, without returning an unmanageably large amount of irrelevant information. Care must be taken not to introduce bias during the literature search (for example, by accidentally searching only sources that tend to report significant findings). Therefore, the *design of a search strategy* needs to be developed thoughtfully with the help of an information specialist experienced in systematic review searches and documented in the protocol.

A comprehensive search strategy should:

- be guided by the primary question, e.g., in the selection of search terms
- minimize potential sources for biases, e.g., by specifying the information sources to be searched
- be in-line with the pertinent inclusion criteria, e.g., publication date or language(s) to be considered
- be developed using syntax specific to databases (e.g., MeSH terms in PubMed)
- strike a balance between sensitivity, i.e., the ability to identify relevant evidence, specificity, i.e., the ability to exclude irrelevant information
- be double-checked for appropriateness, e.g., in a pilot phase
- be appropriately documented in the protocol.

Because including population(s) and outcome(s) in a search can render it too complex to be conducted efficiently, these parameters are not usually considered in clinical systematic review searches. However, a search strategy for a toxicology topic can be expected to include the evidence stream assessed, e.g., animal, human, in vitro or mechanistic studies, and often also the toxicological endpoint(s) of interest.

The *search should be implemented* in an objective manner. In this way, some *biases*, e.g., inclusions of studies known to the authors, or supporting the authors’ view (frequently observed in traditional narrative reviews) can be minimized. Furthermore, authors need to be especially aware of issues such as publication bias, i.e., systematic differences between the findings of published and unpublished research, selective outcome reporting, time-lag bias (time of publication depending on the results), citation bias [(non-) citations driven by the results], gray literature bias (publication in gray literature depending on the results) or multiple publication (Song et al. 2014). It remains to be explored which biases play a role for toxicological systematic reviews.

Some biases can be minimized by an appropriate choice of *sources to search*. Searches will be most efficient in bibliographic databases such as PubMed, EMBASE or Toxline, as they offer advanced search options and will in many cases include at least a substantial proportion of the relevant evidence. A BIOSIS preview, which inter alia includes proceedings, provides access to a certain proportion of gray literature. To determine which sources to search, review teams may wish to contact other experts in the topic of interest, such as research groups or manufacturers. Citation searching may be necessary to complement the database search using tools such as science citation index (SCI) or SCI Expanded, as e.g., implemented by SciSearch or the Web of Science or Scopus. These tools can be used to support the identification of relevant backward citations, i.e., references in eligible studies, as well as forward citations, i.e., later studies referring to an eligible study. It should clearly be stated what level of detail will be required for inclusion, e.g., if posters or abstracts will be considered.

In addition, other sources of evidence may be considered, including general search engines (e.g., Google Scholar), subject-specific or regional databases (e.g., National Toxicology Program study databases), or dissertation and thesis databases.

Gray literature—here defined as material either unpublished or not controlled by commercial publishers, often differing in form, e.g., not a scientific article or report, and the way it is available, i.e., not in journals or databases—may comprise a variety of sources, such as government reports, theses, dissertations, conference proceedings, regulatory databases, case reports or interest group media, such as websites. Gray literature is potentially as important for toxicological systematic reviews as it is for those in the clinical field, but with different emphasis. While in the clinical field gray literature has been shown to include more ‘negative’ results (Hopewell et al. 2007), no evidence on any possible pattern in gray toxicological literature is available. However, it can be expected that it will be associated with intellectual properties. Therefore, some of the original toxicological studies might not be readily accessible for the review team since these are owned by the study sponsors. It is essential to clearly define the gray literature sources to be searched and the type of information to be included (experimental studies, case studies, collection of information (e.g., on websites)). Note that with the new channels of scientific communications, i.e., all forms of web publishing, including blogs, newsletters and websites, the definition of gray literature is currently undergoing adaptation that potentially may require a review of the use of this term in the context of systematic reviews.

Proper *documentation* of the search is required to allow its replication. While the overall strategy design is reported in the protocol, the database-specific search strategies should be included in an appendix to the review. Consequently, the exact strategies should be stored electronically with the date of the search and the number of unique records found. This information should be reported in the first step of a study flow diagram.

The major challenge of the evidence search for toxicological systematic reviews will be to identify the right sources, including gray literature sources, so that the vast majority of relevant evidence is identified. In addition, it will be crucial to provide the means to conduct searches balancing sensitivity and specificity. This could, e.g., be improved by better annotation of the toxicological literature using a widely agreed terminology/ontology. Another issue is that information specialists are often not very familiar with toxicological evidence and databases.

5. Selecting the evidence
  Eligibility criteria
  Selection process
  Documentation

Literature searches usually yield thousands of records. Many of these will either not be relevant or will have specific characteristics that disqualify them for evidence synthesis. To prevent subjectivity in the evidence selection, systematic reviews include definition of *eligibility criteria* that are used to identify the proportion of relevant studies that will ultimately be included in the systematic review. Driven by the review question, it is important to define the criteria as unambiguously as possible to allow consistent interpretation and application by the assessors. To minimize biases and to ensure reproducibility of the process of selecting the studies, the eligibility criteria need to be defined in advance in such a way that the scope for subjective judgment is reduced to a minimum.

Generally, the eligibility criteria for a systematic review will address the pivotal aspects of the framed question, i.e., in case of PECO, its four defining elements. Depending on the scope of the systematic review, the population may refer to a human study population (e.g., in terms of sex, age, geographical region); to animal species, strains and other characteristics for in vivo studies; or to cell source(s) for in vitro studies. Exposure criteria may address the preparation of the doses/concentrations or the administration/treatment (route, characterization, stability, frequency, duration, optimal treatment window, etc.) scheme. Criteria related to the comparison will specify requirements related to experimental controls (e.g., for animal and in vitro studies, the need for a negative or vehicle control) and aspects like randomization and blinding. The eligibility of outcomes needs to be carefully considered in order not to bias the review. Systematic exclusion of outcomes may bias the results, such as excluding evidence that opposes the effect determined by the included outcomes. By contrast, inclusion of a wide range of outcomes may result in a spectrum too broad for a meaningful synthesis, e.g., when considering all types of developmental effects. Other study characteristics that may be used as eligibility criteria include the animal species, reporting of required data and—although often discouraged—the language and date ranges. Studies’ internal validity/risk of bias may be used to exclude studies. However, this has the potential (a) to discard too much evidence perceived low internal validity that may nevertheless be useful and/or (b) to inject a substantial risk of bias, as studies first need to undergo detailed assessment, which may influence reviewers. Therefore, the internal validity of studies is usually accounted for in a sensitivity or subgroup analysis later in the review process.

Another bias may be introduced at this stage of a review if the reviewers’ knowledge results in the formulation of eligibility criteria potentially biased to meet the experts’ expectations. A further potential pitfall at this stage is random error associated with reading and reviewing records.

The *selection process* should be described in detail in the protocol. It should specify the qualification and/or training of the reviewers and how the quality of the selection is controlled (usually by independent duplicate review, i.e., requiring that two reviewers independently carry out the selection, with a procedure to resolve disagreements). In addition, it should provide instruction to document the selection in a way that allows its reproduction. The selection is usually carried out in two stages. First, all identified records are screened, e.g., on the basis of title and abstract, to exclude obviously irrelevant records. Although reviewers should be conservative, and when in doubt, not to exclude studies, screening substantially reduces the number of records. Rejected studies will either be completely off-topic or fail to meet one or more eligibility criteria. These rejections should be clearly documented including a justification. The second stage involves retrieving full reports of the remaining records and determining their eligibility. The task of obtaining full study reports poses challenges to the review team, as both the process and eligibility assessment may be time consuming. For toxicological issues, reports may be scientific articles, but can also be study reports usually in the possession of the study sponsor or conducting agency, which may be difficult to obtain.

Duplicate records should be identified and excluded, usually before the eligibility is evaluated. While some duplicates may be straightforward to identify (e.g., identical records retrieved from different sources), especially when using reference managing software, or other software designed to facilitate systematic reviews, others may be very difficult to detect. Duplicates may be especially hard to detect when only parts of the data have been duplicated.

Researchers may want to consider the appropriateness of the selection process in a pilot exercise. Therefore, a representative subset of studies identified through the literature search should be selected. To assess the reproducibility and appropriateness, two reviewers should independently apply the selection criteria. This practice can identify and remedy ambiguities both in the eligibility criteria themselves or in the way the reviewers interpret them.

Detailed *documentation* of the decision(s) made in the selection process is essential for the transparency of the review. Reviewers’ assessments should be captured, as well as the solutions in case of disagreements. The reasons for exclusion of records at the screening stage should be documented in a dedicated place, where distinguishing the irrelevant cases from the cases that failed eligibility criteria is considered sufficient. Similarly, exclusion at the full-text level should be documented. All full texts retrieved should be kept in a database.

**Fig. 2:**
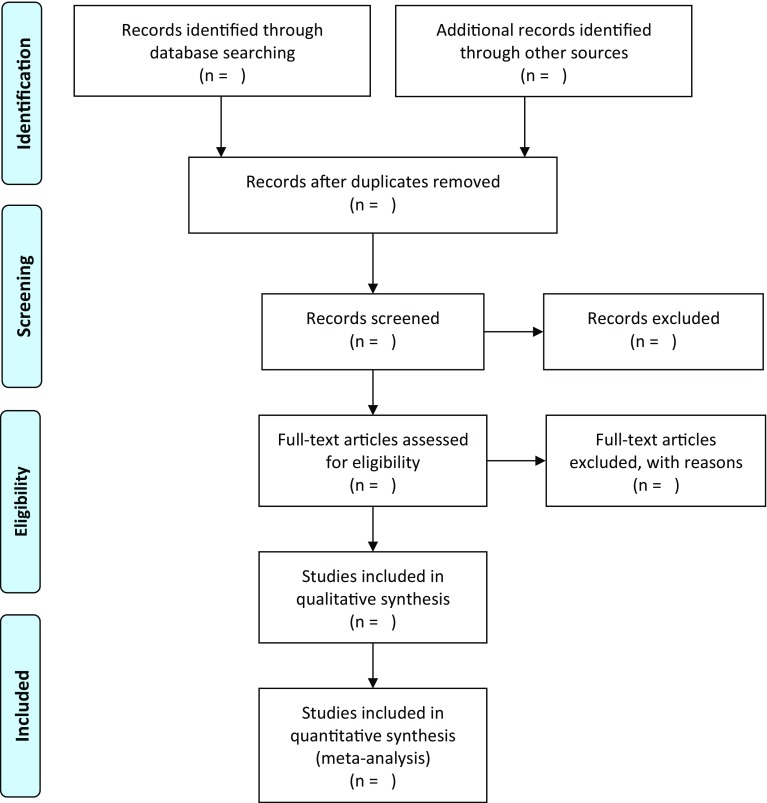
Flow chart of the study selection (from Moher et al. 2009) (screening is based on title and abstracts)

A widely accepted and valuable tool for summarizing the selection process is the PRISMA statement flow chart on study selection presented in Fig. 2 (Moher et al. 2009). Koustas et al. (2014) provide a practical example of its use for a toxicological systematic review.

At this stage, it may be most challenging to learn to handle the possibly vast amount of identified records and appropriately document the selection process. Software applications are available and will likely allow reviewers to implement an efficient and transparent record management. Tsafnat et al. (2014) provided an overview of informatics solutions to support various processes of a systematic review and the systematic review toolbox contains more than 100 software tools for a broad range of systematic review task (http://systematicreviewtools.com/advancedsearch.php).

6. Extracting
  Data to collect
  Data extraction process

Data extraction is the process of collecting relevant information from the full-text version of selected studies for the subsequent data summary and analysis steps of a systematic review. Thorough planning of the extraction is required to minimize biases, reduce human errors, and allow for reproducibility. A priori planning of tables and figures to be included the final report can help ensure that all data relevant for the intended analyses will be collected.

Proper documentation, the use of user-friendly extraction tables or software, and a piloting exercise (see below) will maximize reproducibility of the extraction. Striking the right balance between over- and under-extracting is the key, as failing to collect relevant data may require additional reviews of the full texts of all eligible studies, while extraction of irrelevant data will unnecessarily consume valuable time and resources.

Relevant information includes study characteristics, information pertinent to quality assessment, and study results to be synthesized and meta-analyzed, if needed. *Data to collect* for each study must be defined in advance, and include:

- eligibility, including reasons for both inclusion and exclusion (see above)
- data for quality/risk of bias assessment
- study characteristics (regarding all components, e.g., PECO)
- results
- other information, such as funding source, study authors’ conclusions or if study authors were contacted.

Collected data need to be augmented with general information, e.g., reviewer ID and date of extraction, and with unambiguous study identifiers, such as the citation and a study ID. For some cases, e.g., when only study types with a well-harmonized way of reporting are considered, it is efficient to also extract the data required for the study quality/risk of bias assessment, as described in the next step, during this step. For fields with lesser standardization such as toxicology, it may be more prudent to conduct three different extractions—one for the study characteristics, one for the quality/risk of bias assessment, and one for the results—as each of them requires a different focus and level of interpretation by the extractor.

Usually, most data should be extracted from study reports that allow the data source to be traced. Some journals have included the raw data as supplementary material. In some cases, the funding agencies have a requirement that raw data are submitted to a central database. In some cases, the review team may decide to seek clarification or missing data by contacting study authors. As such obtained information will not be directly accessible to others, it needs to be explicitly annotated. Moreover, this procedure creates the risk of introducing bias, as only some authors will respond. Author response rate should be reported in the final review.

The *data extraction process* is greatly facilitated using electronic data extraction forms. Various nonspecific (e.g., spreadsheet or database application) or specific (e.g., DRAGON, HAWC, RevMan, or DistillerSR) software solutions are available. The extraction form, a sample of which should be included in the study protocol, compiles the relevant data for the review in a clear and unambiguous manner. A clear structure and features such as pre-specified entries increase efficiency as well as user-friendliness, which in turn will reduce human error. It is important to design an efficient extraction process, e.g., using pre-defined lists of values for specific information to be extracted, and by minimizing free text fields.

The extraction itself is a time-consuming process and standardized approaches will help to decrease burdens. Certain study document formats of studies, e.g., PDFs, can facilitate the process by allowing electronic searching. Dual independent review by trained review authors is strongly recommended, while extraction by one reviewer and quality control by a second may be acceptable. Consistency and reproducibility of extraction can be evaluated by a piloting exercise with some representative studies; experience shows that this often leads to modifications of the form. A process to resolve reviewer disagreements should be specified at the outset.

Also, at this stage, efficient and transparent data management might pose the biggest challenge. In our experience, the above-mentioned software solutions allow for efficient and transparent data management.

7. Assessing the evidence
  Terminology
  Internal validity/risk of bias
  Reporting quality

In a systematic review, the quality of the individual pieces of evidence is assessed systematically. In this context, some authorities, especially Cochrane, purposefully avoid use of the term ‘quality,’ owing to the potential for misunderstanding. Consequently, this section briefly introduces the *terminology* used in this context

The term ‘methodological quality’ or ‘study quality’ can refer to study validity as well as to other methodological criteria such as ethical approval and reporting or lack of power (Krauth et al. 2013). An individual study’s validity is composed of its external validity, or relevance, i.e., the extent to which a study provides a correct basis to be generalized to other circumstances, and its internal validity, which is concerned with the reliability of the study itself, regardless of whether it is relevant to other circumstances. A study is internally valid if the differences in results observed between the experimental groups can, apart from random error, be attributed to the intervention under investigation. Certain characteristics of a study may threaten its internal validity, namely if these characteristics introduce systematic differences between the experimental groups other than the intervention of interest. These differences may result in either systematic over- or underestimation of the true effect size, i.e., bias. However, the actual bias (magnitude and direction) in a study can usually not be assessed. Therefore, the term ‘risk of bias’ (RoB) is now widely used in the clinical field to assess the degree of bias susceptibility of a study.

Given that we do not have space to discuss all aspects of methodological quality and given that internal validity/RoB is considered to be a crucial element of quality assessments in toxicological reviews (Krauth et al. 2013; Lam et al. 2014), we will focus in the remainder of this section on internal validity/RoB. However, external validity and possibly other quality aspects of toxicological studies are also important and should be evaluated systematically. Samuel et al. (2016), who provided a systematic compilation of available approaches for assessing methodological and reporting quality of toxicologically relevant studies, present a good starting point for exploring study quality more broadly.

The *internal validity/RoB* assessment evaluates the extent, to which study conduct may have introduced systematic error (i.e., bias) into its results and/or interpretation. This assessment is ultimately one of the several factors that determine the confidence in the systematic review results. The various biases that may affect a study’s internal validity can be assigned to one of the six bias types:

- Selection bias refers to systematic differences between baseline characteristics of the groups that are compared. In experimental studies, this type of bias can be reduced or prevented by randomized allocation and allocation concealment.
- Performance bias refers to systematic differences between groups in the care that is provided, or in exposure to factors other than the interventions of interest. It can be minimized by, for example, blinding researchers and caretakers or by randomizing the order in which the groups receive the experimental exposure.
- Detection bias refers to systematic differences in the way the outcomes are assessed, e.g., when outcome assessors are aware to which experimental group the subject/specimen being assessed belongs. It can be avoided by appropriate blinding and randomization of the outcome assessment.
- Attrition bias refers to systematic differences between the experimental groups in withdrawals or drop-outs from the study. Withdrawals or drop-outs lead to incomplete outcome data. Because the outcomes of the study can only be based on the available data, the reported outcomes may not reflect the true effect of the intervention. Attrition bias can be taken into account by detailed reporting of the number of withdrawals/drop-outs per experimental group and the reason for withdrawing/dropping out.
- Reporting bias refers to systematic differences between reported and unreported findings. For instance, in a published report, those analyses with statistically significant differences between intervention groups, are more likely to be reported than non-significant differences. This sort of ‘within-study publication bias’ is usually known as outcome-reporting bias or selective-reporting bias. The risk of this type of bias can only by assessed if protocols for primary studies are registered or made publicly available before the data analysis. For animal studies, for example, such registration is still highly uncommon.
- Other biases: there may be other sources of bias that are relevant only in certain circumstances or for particular study designs. It is up to the review authors to judge whether for the studies that will be included in their systematic review other factors are likely to cause structural underestimation or overestimation. However, to prevent bias in the application of the criteria for this sixth type of bias, these criteria should be prespecified and clearly defined in the protocol, preferably with an explanation why the criterion in question is likely to reflect an actual risk of bias.

It must be kept in mind that the method for appraising study validity by assessing risk of bias was initially developed in the clinical field, at first for randomized controlled trials. While some of the concepts can directly be transferred to toxicological systematic reviews, e.g., reporting bias, others need to be adapted. Furthermore, for some (aspects of) study types, e.g., in vitro studies on toxicological mechanisms, potential “threats” to internal validity need to be identified.

The RoB assessment is based on specific questions that are defined in advance to address the various bias types. The utility of these questions strongly depends on the kind of evidence to be reviewed. When dealing with human toxicological data, it is possible to adopt the clinical approaches (see e.g., Johnson et al. 2014). With some modifications, the approaches used in clinical systematic reviews have been used for animal experiments, including pre-clinical (see e.g., Wever et al. 2012) and toxicological studies (see e.g., Koustas et al. 2014). For pre-clinical studies, a risk of bias tool has been proposed (Hooijmans et al. 2014a) that focuses on one or more domains for each bias type, e.g., performance bias is addressed by the domains of ‘random housing’ and ‘blinding of caregivers.’ The proposed tool comprises ten specific questions, such as ‘Was the outcome assessor blinded?’ or ‘Are reports of the study free of selective outcome reporting?’ Half of the tool’s questions were in agreement with the Cochrane risk of bias tool (Cochrane handbook) stressing that much can be learned from the role model of clinical systematic review practice. This tool could also be applicable to systematic review of toxicological animal studies. Guidance or examples of risk of bias assessment for in vitro studies are, to our knowledge, not yet available, but the NRC report (2014) addresses this aspect in some detail. Ongoing efforts in toxicology to improve existing scoring systems may be helpful in the future (Segal et al. 2015).

It is important to predefine answers to the questions used to evaluate the risk of bias for each outcome, e.g., low, high, no or unknown risk of bias. This produces results that lend themselves to straightforward summary and improves the consistency and reproducibility of answers from different reviewers. The answer spectrum to the questions should be harmonized across questions as much as possible and should be transparent and clearly described to foster reproducibility. In this process, guidance and/or examples for the answer choices for each question further reduces reviewer disagreement. In addition, answers should be justified, which will help resolving reviewer disagreement.

**Fig. 3:**
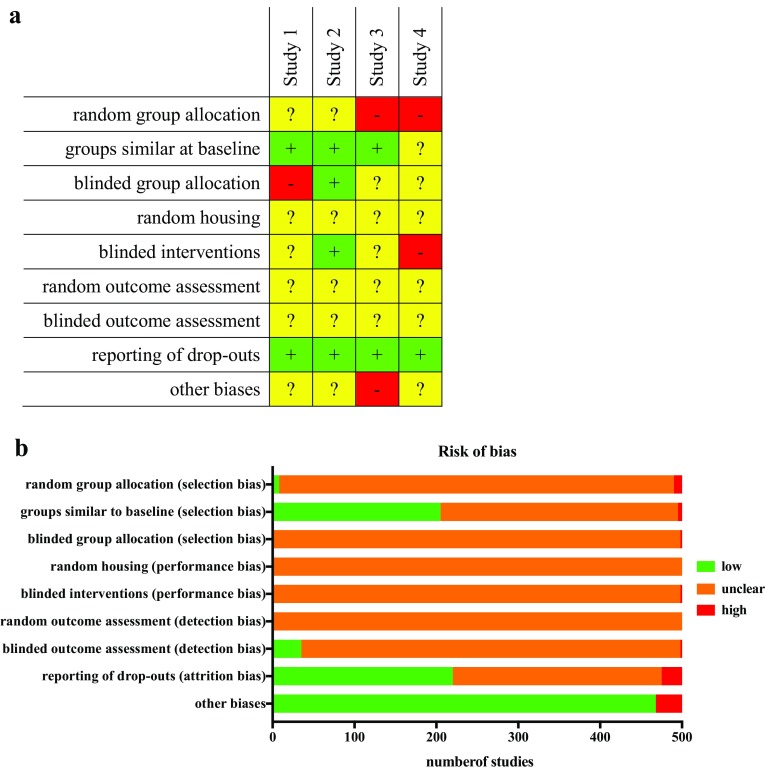
Representative summary table (**a**) for the risk of bias assessment [*green cells* with (*plus*): low risk of bias; *yellow cells* with (*question mark*): unknown risk of bias; *red cells* with (*hyphen*): high risk of bias] and representative summary (**b**) of risk of bias analysis across studies (reproduced from Wever et al. 2015)

The results of the risk of bias assessment for individual study should be reported, e.g., as a tabular matrix of risk of bias questions and included studies (see, e.g., Fig. 3a). Such a matrix facilitates the summary of the risk of bias for each question across all included studies, which is often displayed as a bar chart (see Fig. 3b). In addition, the rationales leading to this assessment should be available. These summary data are to be considered in the synthesis of the results, either narratively or by meta-analysis.

The importance of the various bias domains or questions varies depending on the outcome. For example, detection bias is likely to be less important, i.e., will more unlikely result in bias, for animal studies with death as the primary outcome. Detailed discussions of the applicability of certain domains to toxicological animal studies are available (see e.g., National Research Council (NRC) (2014); National Toxicology Program (2015)).

For all potential sources of bias, it is important to consider the likely magnitude and the likely direction of the bias. For example, if all methodological limitations of studies were expected to bias the results towards a lack of effect, and the evidence indicates that the intervention is effective, then it may be concluded that the intervention is effective even in the presence of these potential biases.

General lack of scientists’ awareness of the risk of bias concept in the toxicological community results in reporting that omits details important for risk of bias assessment. This may seriously hamper assessing the actual risk of bias of the included studies and therefore of the systematic review. Guidance to improve *reporting quality* is available for toxicological animal studies, notably the ARRIVE guidelines (Kilkenny et al. 2010) and others (Hooijmans et al. 2010; Landis et al. 2012). However, harmonization of guidance and focus on potential use in systematic reviews is required. As more toxicological systematic reviews are conducted, their authors will identify which aspects need to be reported (or not), possibly creating an iterative feedback loop motivating authors and even journals.

With regard to use in a systematic review, inadequate reporting per se does not reduce internal validity, but is an obstacle to the assessment of this validity. It remains to be seen if the solution of contacting authors to retrieve missing information is a feasible and useful approach in toxicology. This solution is often applied in the clinical fields, but is generally not very successful because of low response rates. An approach that is likely to work better is education in and adherence to reporting standards, with proper enforcement by journals.

A major challenge for the community is both to find agreement on the importance of the various potential quality (including risk of bias) criteria and to support their importance by empirical evidence as well as determining how best to integrate these measures into developing and supporting conclusions. A good example of a type of bias of which the presence and importance in toxicology is still a topic of debate is sponsorship bias (or funding bias). Although there have been studies that pay attention to this issue (e.g., Wandall et al. 2007; Bero et al. 2015), it has not yet been investigated systematically whether a similar type of bias exists in toxicology.

8. Analyzing data
  Planning the analysis
  Narrative analysis
  Meta-analysis
  Heterogeneity, sensitivity analysis and reporting bias

It is important that the *analysis is planned* a priori, in the context of the review question. To prevent selective outcome reporting, the protocol should describe the planned analysis as precisely as possible, notably the outcome measures to be analyzed as well as the ways to deal with heterogeneity (statistical model, subgroups). The data type (binary, ordinal or continuous) of the measured effects to be analyzed will inform the analytical methods. Because it is not always easy to anticipate all details of the analysis steps, the analysis section of a systematic review protocol sometimes has to be revised once the analysis has been started. It is important that these revisions are clearly indicated and justified both in the protocol and the published systematic review. Consultation with a statistician is strongly recommended, particularly if the data analysis takes the form of a meta-analysis.

A *narrative analysis* (or narrative synthesis) is a descriptive summary of the included studies’ results. It is an essential ingredient of any systematic review that should provide sufficient detail, usually using tables.

A *meta-analysis* is a structured quantitative analysis of outcome data from comparable studies leading to a quantitative summary of results. Note that a systematic review does not have to contain a meta-analysis. If, for instance, the number of studies is too low, the outcome measures vary substantially between the included studies or the studies are too dissimilar in design, a meta-analysis does not make sense. The benefits of a meta-analysis include increased statistical power and improved precision in the estimation of an effect. However, the reliability of the results of a meta-analysis depend on the reliability of the included studies. If a meta-analysis contains many low quality studies (e.g., a high risk of bias), the results can be misleading.

While meta-analysis is the predominant approach in the clinical field, it still has to be explored to what extent and to which type of toxicological review questions meta-analytical techniques can be applied (Goodman et al. 2015). Possibly, lessons can be learned from the experiences made when adapting and applying meta-analyses to pre-clinical animal studies (Vesterinen et al. 2014, Hooijmans et al. 2014b). Meta-analysis of human epidemiological studies has been applied to assess neurobehavioral effects of metal or organic solvent exposure in an occupational setting (e.g., Goodman et al. 2002; Meyer-Baron 2005). Also, unique to toxicology is that many assessments involve quantitative characterization of hazard via toxicological benchmark values, such as daily reference doses or occupational limit values—values that the process of systematic review can support, but the approach for doing so is not yet clear.

Heterogeneity in a meta-analytical context refers to statistical heterogeneity, i.e., variation in the results of studies greater than would be expected from chance alone. This heterogeneity may be caused by several types of differences between the included studies. Such differences may be present in study characteristics such as the population, e.g., when different species, strains or cell lines are used; the exposure, e.g., when various exposure routes or durations have been used; the control, e.g., when different solvent vehicles were employed; or the outcome. Furthermore, study designs and methodological factors, as addressed in the risk of bias assessment may vary between included studies, potentially leading to heterogeneous effect sizes.

The protocol should describe how heterogeneity will be identified and dealt with. An important method of exploring the sources of heterogeneity is performing subgroup analyses, in which the studies included in the meta-analysis are split according to basic characteristics that might lead to differences in effect. For example, it might be of toxicological interest to study in a subgroup analysis whether studies that only used a negative control had different results compared to those employing a solvent control and whether these differences (partly) explain the heterogeneity found in the analysis. Subgroup analyses need to be pre-specified in the protocol to prevent selective reporting and should be limited in numbers to allow meaningful interpretation and to keep control of the multiple testing.

To assess the robustness of the results of the systematic review/meta-analysis, i.e., the extent to which these results depend on the decisions made during the review process, a *sensitivity analysis* can be performed. Although a major aim of systematic reviews is to be as objective as possible, in the conduct of a review decisions have to be made that to some extent depend on subjective preferences. Examples of such ‘subjective’ decisions are the definition of a numerical value (e.g., NOEL vs LOEL) or the choice of statistical methods (e.g., Chi-square test vs Fisher’s exact test). Documentation and justification of these decisions will ensure transparency. In a sensitivity analysis, however, the impact of these decisions can be shown. Such an analysis studies whether the overall results would have been different, if another choice had been made, for example, if a LOEL rather than a NOEL had been used. If the overall results are the same, the conclusions of the meta-analysis are more robust.

In the clinical field, *publication bias*, i.e., bias in the published literature because studies with neutral or negative results are less likely to be published, is a frequently suspected problem. Tools that provide an indication of the presence and/or impact of publication bias, such as funnel plots and trim-and-fill analysis, have been developed. Moreover, the practice of prospective registration of trials, which makes studies traceable, has been established as a countermeasure.

It will be interesting to see whether and in what way meta-analyses can and will be applied in toxicological systematic reviews, whereas a major challenge will be to grasp publication bias in toxicology, i.e., the frequency, the direction and the causes. In the field of clinical studies, what counts as a positive (treatment effective), a neutral (treatment no effect) and a negative result (treatment harmful) is relatively straightforward and similar for all stakeholders involved. In toxicology, it is not that simple. It is even conceivable that publication bias may be present in both directions; while some may not publish unexpected negative results, others may not publish unexpected positive results. In addition, investigation of the external validity of subgroups, e.g., animal species, by a subgroup analysis, which has been used, e.g., by Lalu et al. (2016) for a pre-clinical systematic review, may present a valuable approach for toxicological questions. Consistency in results over subgroups may increase external validity, while inconsistencies may flag the need to identify the evidence most relevant to the review question.

9. Interpreting the results
  Evidence streams
  Confidence in a body of evidence
  Integration of evidence streams
  Conclusions

The interpretation (or synthesis) of the results found and the conclusion of the systematic review should be clear, precise, and comprehensive in light of the review question. The components of this final section of the systematic review should be presented in a way that can be easily understood by scientists, the public, and decision makers.

In toxicology, often data from the various so-called *evidence streams*, i.e., sets of studies representing the same type or level of evidence, e.g., human (observational) studies, animal studies, in vitro or mechanistic studies, need to be integrated. For methodological reasons, it is advisable to conduct systematic reviews for each evidence stream separately. Consequently, interpretation will have to be done at at least two levels; for the individual systematic reviews/evidence streams and for the combination of all evidence streams.

Within each systematic review, the interpretation should be carried out for the so-called bodies of evidence, i.e., sets of studies of the same type or level of evidence grouped by outcome measure. The interpretation is qualitative and aims at determining the confidence (or certainty) in the evidence. The confidence in the evidence expresses the level of certainty that the findings from a group of studies reflect the true relationship between exposure to a substance and the outcome measure in question.

There is no consensus yet on the details of how the *confidence in a body of evidence* should be determined in the field of toxicology. For systematic reviews for environmental health assessments, Rooney et al. (2014) proposed a system for rating confidence in the body of evidence based on GRADE, where GRADE stands for: Grading of Recommendations Assessment, Development and Evaluation (Guyatt et al. 2011; Balshem et al. 2011). The GRADE approach was developed for healthcare systematic reviews. It starts by setting an initial level of confidence depending on study type, e.g., randomized controlled trials start high and observational studies start low. This initial level of confidence may be decreased or increased if certain attributes are present. Attributes that can reduce the confidence in the body of evidence include overall risk of bias, publication bias, imprecision, inconsistency and indirectness, whereas characteristics such as large effect sizes and a dose–response gradient may increase the confidence. The outcomes of this grading process inform a rating of the final confidence in body of evidence, which will guide the conclusions. It is acknowledged that this rating may be subjective. However, the rating is considered helpful as it increases the transparency of the final conclusions.

The approach developed by the National Toxicology Program (2015), and the approach used by the Navigation Guide (Woodruff and Sutton 2014), are similar to the original GRADE approach, but use slightly different criteria for setting initial levels of confidence and for upgrading and downgrading. For example, in the Navigation Guide, observational studies start at a moderate rather than a low level of confidence and NTP uses consistency across species as an extra-upgrading criterion. However, it is re-emphasized that there is not yet consensus in the toxicology field regarding this approach.

Ideally, the confidence in the different bodies of evidence (i.e., per outcome) should be integrated into a confidence across outcomes for the evidence stream in question.

As indicated above, a complexity of toxicology, in contrast to healthcare, is the potential need for *integration of evidence streams*. It has been proposed to distinguish the following three evidence streams: ‘human’; ‘animal’; and ‘mechanistic/in vitro’ (National Toxicology Program, 2015). It remains to be seen if this categorization of evidence based on the test species/system is sufficiently coherent, as mechanistic evidence may also be derived from animal studies and in vitro studies may provide other information than mechanistic. However, mechanistic understanding is essential in the assessment of external validity, especially of non-human evidence. Although the integration of these evidence streams is not part of the systematic review process (it is more about the integration of the results of different systematic reviews), it is desirable that this process is conducted in a structured, transparent and pre-specified way.

The methods to integrate different evidence streams are even less established than the methods to interpret and rate bodies of evidence. An important reason for this is that, at this level, not only the confidence in each evidence stream may be important but also its relevance, e.g., for the human exposure of interest, or external validity.

Questions that arise at this level are: Should all evidence streams get the same weight or should the evidence streams be weighted by their external validity, for example, the evidence from mechanistic in vitro studies, by definition, have a lower weight because these data might have lower relevance for the human situation? This is especially relevant, since more Adverse Outcome Pathways are available as well as data from programs like US EPA ToxCast™ and TOX21, providing a wealth of data on a molecular level. For example, if the human evidence stream has a low confidence level (e.g., because these studies contain many confounding factors) and the mechanistic stream has a high level (e.g., because it consists of well-designed and well-conducted experimental studies with very similar results), should the latter get more weight than the former?

NTP has developed a framework that translates confidence ratings per evidence stream into evidence of health effects, which is then used to integrate evidence from human and animal studies into a hazard identification conclusion. The mechanistic evidence is used to support the decision to turn the initial hazard conclusion into a final hazard conclusion. Rhomberg (2015) has suggested a different approach for integrating data from different evidence streams, called a hypothesis-based weight-of-evidence approach. However, a standardized approach does not yet exist.

The *conclusions* of the systematic review should detail the implications of the findings. These may vary depending on the study question(s), but should not go beyond the review scope. For example, in cases where a specific hazardous property of a substance was investigated, the conclusions should make a clear statement about that hazard. Other potential topics to address in the conclusions section include the implications for research, e.g., how to address identified data gaps or how to solve methodological issues, and the reflection on limitations in design and conduct of the review itself.

The data analysis is posing two main challenges. It remains to be seen how to determine to best adapt the GRADE approach to toxicological questions, as discussed by Morgan et al. (2016), or whether an alternative system is more appropriate. In addition, further methodological discussions and case studies are needed to explore how to integrate bodies of evidence within an evidence stream and across evidence streams.

10. Reporting
  Basic requirements
  Reporting elements
  Presentation of findings

Regardless of the specific form of a systematic review report, some *basic requirements* exist that are applicable to the reporting of toxicological findings in general. General authorship rules apply, such as identifying a corresponding or lead author. These decisions are likely to be made as part of protocol development and establishment of team members and roles. Of particular importance to systematic reviews, conflict of interest statements for all authors are required. Most, if not all, systematic reviews will be reported in English. Correct spelling and grammar, and clear, concise language should be used, keeping in mind that audiences will often include non-toxicologists. Toxicological systematic reviews should undergo peer review and should be made publicly available.

Systematic reviews can be written up in the form of a stand-alone report (perhaps published on a website) and/or a publication in a peer-reviewed journal. While these two reporting forms may differ in length due to journal restrictions, both should cover essential *reporting elements*. If important information cannot be included in a journal publication, this should be made available elsewhere, e.g., as supplementary documentation, with a level of detail that allows reproduction of the review, or through data repositories.

Systematic review reporting guidelines are available for human studies, especially for intervention studies, but there is less established information on reporting toxicological systematic reviews. However, the structural elements that are generally recommended for systematic reviews provide valuable guidance relevant for toxicological systematic reviews. For example, the PRISMA (Preferred Reporting Items for Systematic Reviews and Meta-Analyses) statement is a well-recognized source (Moher et al. 2009). Its checklist elements include the following:

- Title
- Structured abstract/executive summary
- Introduction including the rationale and the review question
- Methods according to the review protocol including evidence search and selection, data extraction, quality assessment and data analysis
- Results
- Discussion summarizing results and including a conclusion
- Funding sources

This checklist, with minor amendments, has been adopted by others (Sena et al. 2014; Whaley et al. 2016). In addition, commissioning bodies or organizations conducting systematic reviews may have or may develop specific reporting requirements.

The results should *present findings* in a clear and structured manner, using tables and figures. For example, two tables may be derived from the extracted study data: one for the characteristics of included studies (such as authors and year of publication, source, study design aspects, essential components (e.g., PECO)), as well as one for the study data and results. The most important findings should be summarized narratively, and if possible, complemented by a tabular summary. A flow chart on the study selection process (Fig. 2) should be included.

The challenge in reporting toxicological systematic reviews will be to include all information that is relevant to allow independent replication of the review. Meeting such requirements may be facilitated by the use of online repositories for protocols, as well as other reporting materials (e.g., extraction and quality tables), particularly for large assessments.

## Conclusion

This primer is an introduction to the application of the systematic review process to toxicological issues. It is intended primarily for those unfamiliar with systematic reviews, who would like to understand them better and/or conduct their own. It draws on existing guidance from the fields of clinical medicine, environmental sciences, food and feed safety as well as the emerging guidance in toxicology. The existing guidance documents compartmentalize a systematic review into different numbers of individual steps. In this primer, a fine-grained approach to parsing the various review steps was chosen to deliver the information in succinct components. For each step, the most important aspects to be considered are highlighted. To summarize, our framework for a systematic review consists of ten steps and their associated topics:

1. Planning
2. Framing the question
3. Developing and publishing of the protocol
4. Searching for evidence
5. Selecting the evidence
6. Extracting (the data)
7. Assessing the evidence
8. Analyzing data
9. Interpreting the results
10. Reporting

Conducting a systematic review is not a trivial task, often specifically funded as independent studies, especially for healthcare interventions. The process differs substantially from that involved in narrative reviews, which are commonly used in toxicology. Although systematic reviews have clear advantages, such as their explicit methodology and transparency, they require diverse expertise, substantial resources, time and for most questions the availability of sufficient data. Therefore, it is advisable to carefully consider the intentions and aims of a toxicological review to decide on the type of review to be conducted. When a systematic review is not feasible, a narrative review may be justifiable. However, even in such cases, reviewers should consider the feasibility of implementing at least some of the individual systematic review steps. For example, an explicit, clear and unambiguous statement of the review question should always be provided. In addition, some basic elements of a systematic literature search, such as specifying the databases searched, the search date and the search terms and strategy, can be easily implemented. These steps nevertheless increase the transparency of the process of study selection and the search criteria, and ultimately would improve the reproducibility and quality of narrative reviews.

We hope the increased awareness of systematic review will result in a shift towards including more systematic review elements into toxicological reviews. This shift should also help to identify challenges specific to toxicology that do not allow adoption or simple adaptation of available systematic review methodology. We identified some of those challenges and summarized them in Table 3. They may require new solutions compatible with the evidence-based principles of transparency, consistency, and objectivity, which provide the foundation of evidence-based approaches.

**Table 3:** Challenges in adapting systematic review methodology to toxicology

Systematic review step	Challenges specific to toxicology
Planning	Composition of a skilled review team covering all fields of expertise required, especially systematic review experience
	Definition of the role of the systematic review sponsor
Framing the question	Framing of the question in a way that it is amenable to systematic reviews
Developing and publishing of the protocol	Publication of protocols so that they are highly and timely visible to stakeholders and interested parties
Searching for evidence	Identification of sources to be searched, including gray literature sources
	Provision of means to conduct appropriately balanced searches, e.g., by better annotation of the toxicological literature
	Familiarization of information specialists with toxicological evidence and databases
Selecting the evidence	Handling of the possibly vast amount of identified records and appropriate documentation of the selection process
Extracting (the data)	Efficient and transparent data management
Assessing the evidence	Determination of the importance of the various potential quality aspects, e.g., by empirical evidence
	Determination on how to best to integrate quality appraisal results into developing and supporting conclusions
Analyzing data	Exploration of the role of publication bias in toxicology, i.e., the frequency, the direction and the causes
	Exploration of the use of investigating external validity of subgroups
Interpreting the results	Determination of the confidence in a body of evidence
	Exploration of how to integrate bodies of evidence within an evidence stream and across evidence streams
Reporting	Making available all information relevant to allow for independent replication of the review

We recognize that any brief introduction to such a complicated topic will have limitations. Many of the topics addressed above are quite complex, and will require further guidance for complete understanding and practical tools for their implementation in the systematic review methodology. Some experts may question the systematic review steps proposed here, preferring instead an alternate framework. Such issues are only natural, as the application of systematic review to toxicological issues is just emerging. Consequently, there is little direct experience and empirical evidence available to guide these types of systematic reviews, although the knowledge base is rapidly increasing. It is the aim of this primer, and in particular of the challenges highlighted, to stimulate development of tools that facilitate the application of systematic review in toxicology, and to encourage the application of the methodology. The systematic review process will be instrumental in guiding toxicology to a more evidence-based science that is rooted in transparency, objectivity and consistency.

## Glossary^1^

Advisory group: A group of people including representation of relevant stakeholders, with relevant interests, skills and commitment to support the review team, e.g., in key decisions, interpretation and dissemination
Attrition bias: Bias due to systematic differences between treatment and comparison groups in withdrawals or exclusions from the results of a study.
Bias: A systematic error or deviation from the truth in results or inferences. A common classification scheme for biases of individual studies includes selection bias, performance bias, attrition bias, detection bias, while publication bias refers to a set of studies
Cochrane: Cochrane organizes medical research information in a systematic way to facilitate the choices that health professionals, patients, policy makers and others face in health interventions according to the principles of evidence-based medicine
Comprehensive search strategy: The exact terms and their combinations (including Boolean operators) used to search a bibliographic database designed to be sensitive, i.e., not to miss relevant evidence
Confounding: A situation in which a measure of the effect is distorted because of an association between the intervention (or exposure) with other factor(s) that influence the outcome under investigation
Data extraction: Data extraction is the process of retrieving primarily study characteristics and outcome data, out of included evidence sources for further data processing
Detection bias: Detection bias refers to bias due to systematic differences between groups in how outcomes are determined.
Eligibility criteria: Pre-defined criteria are derived from the review question and used to select eligible studies, i.e., those to be included in the systematic review
Evidence-based medicine (EBM): Evidence-based medicine is the conscientious explicit, and judicious use of current best evidence in making decisions about the care of individual patients. The practice of evidence-based medicine means integrating individual clinical expertise with the best available external clinical evidence from systematic research
Evidence-based toxicology (EBT): The discipline of evidence-based toxicology is a process for transparently, consistently, and objectively assessing available scientific evidence to answer questions in toxicology
Evidence-based Toxicology Collaboration (EBTC): Evidence-based Toxicology Collaboration is a collaboration of science regulatory and industry leaders, united in their vision to improve the public health outcomes and reduce human impact on the environment by bringing evidence-based approaches to safety sciences
Evidence stream: Toxicological evidence can be assigned to evidence streams, i.e., sets of studies representing the same type or level of evidence—e.g., human (observational) studies, animal studies, in vitro or mechanistic studies
External validity: The degree to which the results of a study hold true in other settings (generalisability).
Gray literature: Gray literature is material either unpublished or not controlled by commercial publishers often differing in form, e.g., not a scientific article or report, and the way it is available, i.e., not in journals or databases
Heterogeneity: Used in a general sense to describe the variation in or diversity of, participants, interventions, and measurement of outcomes across a set of studies, or the variation in internal validity of those studies. Used specifically, as statistical heterogeneity, to describe the degree of variation in the effect estimates from a set of studies. Also, used to indicate the presence of variability among studies beyond the amount expected due solely to the play of chance
Internal validity: The degree to which a result of a study is likely to be free of bias (systematic errors), i.e., has measured what it had intended to measure
Meta-analysis: The process of synthesizing outcome data from a number of independent studies using statistical methods.
Methodological quality: The extent to which a study is likely to be free of features that reduce trust in the results including internal validity and aspects such as ethical approval, reporting and statistical power
Narrative review: Expert summary of a set of publications from which conclusions are drawn without a detailed and transparent description of what was done to allow reproduction
PECO: The components of population/participants, exposure, control/comparison and outcome that the question of a systematic review of an exposure should cover.
Performance bias: Performance bias refers to bias due to systematic differences between groups in the care that is provided or in exposure to factors other than the interventions of interest
PICO: The components of population/participants, intervention, control/comparison and outcome that the question of a systematic review of an intervention should cover
Protocol: The plan or set of steps to be followed in a study. A protocol for a systematic review should describe the rationale for the review the objectives, and the methods that will be used to locate, select, and critically appraise studies, and to collect and analyze data from the included studies
Publication bias: Publication bias is caused when only a subset of all relevant studies is published. The publication of research often depends on the nature and direction of the study results.
Reporting bias: Reporting bias is introduced when only selected outcomes are reported (applying both to systematic reviews and primary studies).
Reporting quality: Reporting quality describes how well and complete a study is reported, ultimately allowing reproduction
Review team: The team that conducts the systematic review with ideally all the various required areas of expertise represented
Scoping: An approach to searching the literature that can be used to support decisions about whether it is possible or worthwhile proceeding with a systematic review.
Selection bias: Selection bias refers to bias due to systematic differences between baseline characteristics of the groups that are compared.
Sensitivity analysis: An analysis used to test the robustness of findings and determine how sensitive results are to the data that were included and/or the way that analyses were done.
Subgroup analysis: In the context of a meta-analysis subgroup analyses compare the effect sizes of different subgroups of studies/experiments within the included evidence. Subgroup analyses may be conducted as a means of investigating heterogeneous results, or to answer specific questions about particular subgroups (e.g., if the effect depends on sex)
Systematic review: A review of a clearly formulated question that uses systematic and explicit methods to identify, select, and critically appraise relevant research, and to collect and analyze data from the studies that are included in the review
Weight-of-evidence: Weight-of-evidence is a process of taking into account different types of scientific evidence based on the strength and limitations of individual studies, in assessing the validity of a causal hypothesis, usually referring to both evidence syntheses within the individual evidence streams and to the evidence integration across evidence streams
